# Miasm

**Published:** 1860-09

**Authors:** 


### MIASM.

A SHORT word, but it brings sickness and death to hundreds of thousands every autumn; it will bring sickness and death sooner or later to many a reader of this article, but a sickness and death which could have been avoided.

Miasm is the principal cause of nearly every epidemic disease; that is, of every sickness that "falls upon the people," attacks numbers in any community, such as fever and ague, diarrhea, dysentery, cholera, bilious, intermittent, congestive, typhoid and yellow fevers. But it is an avoidable cause of disease. Money and wisely directed efforts can banish it from any locality. All that is needed is to know the laws of miasm, and adapt ourselves to them. The New-Orleans *Bee*, which is one of the very best daily papers in the South, conducted with uniform ability, recently made the following statement on the mystery and invincibility of yellow fever.

"The yellow fever has broken out in New-Orleans under every conceivable variety of circumstances—when the streets were clean, and when they were filthy—when the river was high and when it was low; after a prolonged drouth, and in the midst of daily torrents—when the heat was excessive, and when the air was spring-like and pleasant—when excavation and disturbances of the soil had been frequent, and when scarcely a pavement had been laid or a building erected. If the disease is endemic and indigenous—a point still in dispute—all we can say is, that research, inquiry, and sagacity are baffled in the attempt to trace its origin and develop its causes. It comes without warning, and goes we know not whither.

"Almost the only fixed and undeniable fact connected with the disease is that its prevalence is simultaneous with the heats of summer and that frost is its deadly enemy. From these frank acknowledgments it may be understood how exceedingly limited is knowledge of the subject. Although most deeply interested in it, and although for half a century the most prominent and learned physicians have bestowed labor and investigation upon it, they have failed to establish beyond contradiction and controversy a single fact that would prove of clearly practical utility in guarding against the approach of the destroyer, or in cutting short its ravages."

There is not a material statement in the above extract that is correct. Sufficient is known of the Law of Miasm to make New-Orleans one of the healthiest cities in the Union. Having received our medical education in the South, and lived and practiced there many years afterwards, and especially in New-Orleans, it may be presumed that we speak not altogether unadvisedly. Not to keep the reader waiting, the plan will be first stated and then the reasons given ; a mistake is certainly not impossible.

Dig a basin in the swamp, in the rear of the city, its sides so cemented that no water can permeate them, and let a system of city drainage be instituted which shall end in this basin, to be pumped out from June until frost, and delivered into lake Pontchartrain. The Dutch have drained a young State by a steam-engine. The American ought to be able to reclaim a few acres by the same agency. All details are purposely left out. The city of Louisville, Ky., in our early recollection, was one of the most pestilential spots in the habitable West; by filling and draining it is now one of the healthiest, one of the most beautiful and flourishing of the cities of the great valley.

Miasm is an invisible emanation, an emanation from vegetable matter, dependent always and every where on three conditions, any one of which being absent, its generation is an impossibility. There must be vegetable matter, there must be moisture, there must be heat of eighty degrees Fahrenheit and over, for a considerable portion of each day. These things are known and acknowledged by all scientific writers on the subject, hence it is not necessary to prove them. If then there is no wood or leaves in a specified locality, there can be no miasm generated there; nor can there be, if there is no moisture; nor can there be if there is no heat. No human power can get clear of the heat of New-Orleans, and profuse vegetation is characteristic of that latitude, so that the only practical mode of preventing the generation of miasm there, is to cause a dryness of the surface-soil by means of drainage. The "Mystery" of Miasm, is as plain as the face of day when its laws are known. Dr. McFarland of New-Orleans has not hesitated to publish, that the relative filthiness of the city has nothing to do with its reputed sickliness, in fact, that the filthier parts were the healthiest. The nature of miasm is such that an epidemic may prevail in New Orleans in a very dry summer or a very wet one. One summer may be a dry one, and there be no yellow fever; the next may be a wet one and no yellow fever; the third may be a dry one and yellow fever may be epidemic; the fourth may be a wet one, and the yellow fever may be epidemic, and yet one invariable law of miasm governs the four years, and which, when understood, is plain, even to a child's comprehension. Every man is interested personally and vitally in these statements, whose family lives where autumnal diseases prevail.

Among the phenomena of miasm are these. First. It impregnates the air of the locality, in or very near which it is generated, and to be affected by it, a person must be in that locality, and breathe its air.

A son of Colonel Ellis was attacked with yellow fever at his residence, where the disease had never been known, and there was no other case in the family or in the neighborhood. Just before he died, he was asked if he had been to Philadelphia, where it was then raging in all its fury, he replied, he had not been in the city, had only crossed over to the shipping, but had not gone up into town. It was at the shipping, along the wharves, where it originated, and always does originate. Families do not take yellow fever from yellow fever patients, brought to them several miles in the country, from the city, if they have not been exposed in any other way. To get any prevailing disease a man must go to the locality where that disease exists. None took the National Hotel Disease, but those who entered the building. In Boston, during one of the cholera years, only the inmates of a certain house, in a specified district, were attacked. It was ascertained that in a dark part of the cellar, there was an immense amount of kitchen garbage which had been in the course of daily deposition for many months. The lesson is, that when a disease prevails in a ward of the city, in a certain part of a village, or county, or district, there is a local cause for that disease, in a combination of vegetable matter, heat and moisture, and families should at once be removed, and measures taken to ascertain the source of the evil, and remedy it.

It is a fearful fact, that of each hundred English soldiers in India, ninety-four disappear from the ranks before the age of thirty-five, when from official statements, it is known that "the average standard of health for Europeans in India, would bear comparison with that existing any where else in the civilized world if the known sources of disease were dried up." It is admitted that in forty years, one hundred thousand men might have been saved, if "proper localities had been chosen for their dwellings." This shows that a rich man in retiring from business, should not be guided in the selection of a locality for building or purchase, as a residence by the "prospect," or facilities of getting to town. The determination should be made, according to the known laws of miasm.

The hospitals and barracks, in and near Bengal are now almost useless, having been erected in places utterly unfitted for the purpose, in a hygienic point of view, and yet the cost of their erection to the British government was the enormous sum of sixty-five millions of dollars! A very limited knowledge of the general laws of miasm would have prevented this shameful loss.

Miasm is of a nature so intangible, that no chemical analysis has ever been able to fix it, to take hold of it, to discern it; it is as viewless as the air itself. An atmosphere saturated with it, gives no more indication of its presence than the purest air of the poles. We must therefore judge of its nature and laws, from its effects. The low shores of sluggish rivers, lakes, and ponds, and flat marshes, when exposed to a hot sun, are the great manufactories of deadly miasm. In the heat of the day, this miasm is created with great rapidity, but in the heat of the day it is innocuous, this seems a "mystery," but it is easily explained, when a little more is known. Heat rarefies the air which contains the miasm, so rapidly, that it rises instantaneously to the upper regions of the atmosphere, where it can not be breathed. But the cool of the evening condenses it, makes it heavy, causes it to fall to the surface of the earth, where it is breathed and becomes the cause of various diseases, sometimes of such malignity, as to resemble ailments produced by the most virulent poisons, as in the case of the National Hotel of Washington City three years ago. Hence it is, that in crossing the Pontine marshes near the city of Rome, the traveler is cautioned against spending the night there, as it is almost certain death.

Very recently a gentleman had occasion to pass that way, and contrary to the warnings of his friends in the city, he spent a night there, the hotel-keeper assuring him that there was no danger whatever to strangers; the result was, that he died of malignant fever in a few days.

Very recently a daughter of one of the best and wealthiest families in Brooklyn, having graduated with great honor, visited Rome, but unwittingly exposing herself to the deleterious miasms of the Campagna, "lured by the cool and balmy air of an autumn evening" to breathe its poison, she died. Miasm which is generated by heat, becomes deadly to man only when the atmosphere, by becoming cool, condenses it, makes it heavy and causes it to settle near the surface of the earth, where it is breathed, to remain there until the morning sun begins to warm it up again, rarefy it, and carry it above the breathing-point, and so attenuating it, that if it were breathed it would be comparatively harmless. But when the weather becomes so cool as to deposit the miasm in a layer of a few inches' thickness on the surface of the earth, and the heat of the atmosphere at the surface is under eighty degrees, epidemics cease; hence frost is said to "kill" yellow fever; this is not exactly so, for if a few warm days were experienced the disease would return, would again become epidemic, warming the chilled miasm into life, and generating more of it. If then cold paralyzes miasm, and heat carries it rapidly above, where it can not be breathed, both cold and heat are its antagonisms, but to be perfectly efficient, cold and heat must be continuous, with this distinction cold only paralyzes, heat removes. Heat will disinfect a vessel, where disease is raging, and not a single new case will occur, even after the heat is removed, if the ship has been left perfectly dry inside, because the destructive agent has been carried out of the vessel, and has been scattered in the region of space above and beyond. Several tons of ice will also promptly cut short the disease, more instantaneouly than heat, because it condenses the miasm which is thereby made heavier and settles within a few inches of the bottom of the vessel, but as soon as the ice is removed, the external temperature remaining the same, the miasm becomes rarefied, rises and revives the disease. These facts give rise to apparent contradictions, and which sometimes appear inexplicable. Heat generates malaria, and at the same time renders it innocuous, cold paralyzes malaria, and it becomes hurtless. Again it is thus seen, how a little heat, applied to chilled miasm, may restore to it its violence, while a little cold after heat, brings it down to the surface from above, and gives it its malignity. Hence, at sun-down of an autumn day, it becomes a little cold, and the miasm becomes malign; at midnight it is colder, and it becomes hurtless; at sunrise it becomes a little warmer, and the miasm rises from the earth, and covers it five or six feet deep, to be breathed and to destroy; by midday, the greater heat has carried it again beyond the hurtful point, thus the reader sees that there is no "mystery" in miasm, but that it is governed by laws as fixed as mountains of adamant. If a mill-pond is drained in the fall of the year when the days are hot and the nights cold, just leaving the bottom in a mortary condition, it would bring disease and death in a few days, if not in a few hours, to all who slept on its banks. No traveler dared to sleep at night on the flat damp Isthmus of Panama, in the early days of California travel. But if that mill-pond were flooded a foot or two deep in water, the disease would abate as soon as if there had been a severe frost, because miasm can not be generated and rise through two feet, or even one of water. That is the reason, why the "Swamp," in the rear of

New-Orleans is sometimes perfectly healthy, when the city proper is being wasted by disease, because the swamp is inundated by the spring rise of the Mississippi or by a steady hard wind from the Gulf towards the city, for several days, which we have known to raise the lake so as to flood the swamp, and nearly the entire city, nearly up to the old State-House on Baronne street, where our office was, leaving only a ridge of dry land between that and the river, some eight or ten blocks distant. Thus a very wet summer would keep the swamp flooded so deep as to prevent the generation of malaria; but if not locally wet, on the contrary dry, the Mississippi in its annual rise would do the same thing, as also a steady wind from the Gulf, and if about the time an "overflow" from either cause was subsiding, heavy rains should come, then a fall "blow," the swamps would be kept inundated until frost, and thus the whole city would have been healthier than was known to have been in the memory of the "oldest inhabitant." A very long and severe drought, with no "blows" from the Gulf and no overflows from the Mississippi would make the swamp so dry, that generation of malaria would be impossible. By failing to make these discriminations, it does appear to make no difference whether it is a hot or comparatively cool summer, whether wet or dry, but looking closely into the laws of miasm, order is brought out of confusion, and all things proceed in beautiful harmony.

Five families may dwell within half a mile of a drained mill-pond, upon the damp bottom of which an August or September sun beams with dog-day fierceness, and yet, by the known laws of miasm, only one family will suffer from it; the other four will be as well as usual. First. If a rapid river runs between the pond and the house, there will be no sickness at all. Second. If a thick growth of trees and bushes, only a few feet broad, interpose, there will be no sickness. Third. If the prevailing winds be from the house to the pond, there will be no sickness. Fourth. If the house be on a steep hill, there may be no sickness, because miasm does not cross a wide, rapid river; miasm does not pass a thick wall of vegetable growth; miasm can not travel against the wind; miasm can not ascend a steep high hill. There is no mystery in these varieties, and no complexity, because they all arise from simple, unvarying natural laws.

One year a house immediately on the bank of a mill-pond will suffer; the second year it will escape, because it is a very cold summer; the third year it will escape because it is a very hot summer; the fourth year it will escape, because it is a very wet summer. Why? The wet summer kept the bed of the pond covered over with water a foot or two deep, and miasm could not form. The hot summer made the bed of the pond dusty, and miasm could not form; the cold summer did not give a heat of eighty degrees, and miasm could not form. Here is variety, but uniformity; mysterious to the uninformed, but to the student and observer, it is as clear as the sunlight! Any man may sleep a whole night in the Pontine marshes with impunity, if he close his chamber, and hang ice in it, so as to reduce the temperature below eighty degrees, for then the miasm would be condensed to the surface of the floor; or if he have no ice at all, and keep a fire blazing on the hearth. If, however, he slept on the floor with the ice, he would breathe the miasm in its concentrated form, and suffer for it; in case of the fire protection, he should sleep on the floor, because the heat would carry the miasm to the ceiling. All these points were beautifully illustrated in the famous or infamous Hotel at Washington. The miasm was so deadly, that many intelligent persons asserted then, and believe yet, that it was the result of mineral poison. This miasm was generated from sewers opening into the cellar, and from privies on the ground-floor which were testified to have been so full, that when a person stepped on the floor, the compound beneath spouted up between the planks. On an official investigation it was shown that men, who came into the bar-room for an hour, suffered; a man who came fatigued after a day's travel, and ate a single meal on the lower floor, nearly died, while ladies who had lived in the house all winter wholly escaped. These, however, are but seeming "mysteries." It was winter. The miasm was generated about the ground-floor of the building, where it was comparatively cold, causing the deadly agency to remain condensed and concentrated where it was produced, and by the men who spent there most of their time it was breathed. The ladies occupied the upper rooms and kept their fires steadily burning, which rarefied any miasmatic air that made its way from below, and carried it at once to the ceilings of their respective apartements, while the winter air in all its purity came directly from without, through window crevices, not only diluting the already rarefied miasmatic air, but driving it out by a current through the crevices at the tops of the inner doors especially.

Again. The stomach of a hungry man will drink in miasm with all the greediness of a famished wild beast, the whole blood will be poisoned in an hour, and the drowsiness or stupor of death will steal stealthily over the unconscious victim. Within a month, we met an intelligent young Roman patriot, who had spent many a day in hunting on the Pontine marshes; directing his attention to the subject, he said, that on some occasions, they were forced by laughter and yells, and uproarious songs, and pushing and slapping one another, to keep themselves from going to sleep; he said that to sleep was to die, almost as certainly as if yielded to among the snows of Mont Blanc or of Greenland. On one occasion having had nothing for some hours, to stay the stomach, the foundation for a tedious and serious illness was laid. One other brief lesson may be learned, one which merits annual publication in every paper in the land, and which ought to be kept in a gilded frame above the mantle of millions of dwellings.

As moderate coolness brings the miasm to the surface of the ground, say within its first five or ten feet, the morning and the evening, embracing the hour including sun-down and sun-up, in the fall of the year, are the most dangerous periods of exposure within the twenty-four hours; and yet the atmosphere is so delicious at those times, that to resist being out in it, and breathe it, is impossible but to the few. Hence, the remark has been made in our hearing in New-Orleans, that whenever the evening air seems for a few days to be most delicious, in epidemic seasons, it is taken for granted, that an increase of the disease will certainly follow. Here, the purer the air, the worse the disease! a seeming truth, and a seeming "mystery," but false and clear in the light of a sound information.

If then hunger, or an empty stomach, or, which is the same thing, a weak stomach, ravenously drinks in, as it were, the deadly poison, and the air is most charged with it at sun-down, and sunrise, it clearly follows, that in epidemic times, families should gather around a cheerful fire at sunrise and sunset, and should fortify the stomach against the malignant miasm, before they go out in the morning, with a good breakfast, and at sundown strengthen it with a cracker and a cup of weak tea against the effect of the fatigues of the labors of the day just closing. Families who will conduct themselves in accordance with the principles laid down, can procure a happy exemption from autumnal and epidemic diseases. To do so, however, requires an amount of intelligence, self-denial, and moral courage possessed by the few, but we are not without hope that the knowledge of these things will gradually spread among whole communities, who acting from conviction and principle as to the daily conduct of life, will add a score of years to the average age of men.

